# Diagnostic classification of coronavirus disease 2019 (COVID-19) and other pneumonias using radiomics features in CT chest images

**DOI:** 10.1038/s41598-021-97497-9

**Published:** 2021-09-09

**Authors:** Ning Yang, Faming Liu, Chunlong Li, Wenqing Xiao, Shuangcong Xie, Shuyi Yuan, Wei Zuo, Xiaofen Ma, Guihua Jiang

**Affiliations:** 1 Department of Medical Imaging, Guangdong Second Provincial General Hospital, Guangzhou, 510317 People’s Republic of China; 2 Radiology Department, Xiao Chang First People’s Hospital, Hubei, People’s Republic of China; 3 Majoring in Imaging and Nuclear Medicine, Graduate School, Guangdong Medical University, Guangzhou, People’s Republic of China; 4 Equipment Department, Guangdong Second Provincial General Hospital, Guangzhou, People’s Republic of China

**Keywords:** Biomedical engineering, Medical imaging, Respiratory tract diseases

## Abstract

We propose a classification method using the radiomics features of CT chest images to identify patients with coronavirus disease 2019 (COVID-19) and other pneumonias. The chest CT images of two groups of participants (90 COVID-19 patients who were confirmed as positive by nucleic acid test of RT-PCR and 90 other pneumonias patients) were collected, and the two groups of data were manually drawn to outline the region of interest (ROI) of pneumonias. The radiomics method was used to extract textural features and histogram features of the ROI and obtain a radiomics features vector from each sample. Then, we divided the data into two independent radiomic cohorts for training (70 COVID-19 patients and 70 other pneumonias patients), and validation (20 COVID-19 patients and 20 other pneumonias patients) by using support vector machine (SVM). This model used 20 rounds of tenfold cross-validation for training. Finally, single-shot testing of the final model was performed on the independent validation cohort. In the COVID-19 patients, correlation analysis (multiple comparison correction—Bonferroni correction, P < 0.05/7) was also conducted to determine whether the textural and histogram features were correlated with the laboratory test index of blood, i.e., blood oxygen, white blood cell, lymphocytes, neutrophils, C-reactive protein, hypersensitive C-reactive protein, and erythrocyte sedimentation rate. The final model showed good discrimination on the independent validation cohort, with an accuracy of 89.83%, sensitivity of 94.22%, specificity of 85.44%, and AUC of 0.940. This proved that the radiomics features were highly distinguishable, and this SVM model can effectively identify and diagnose patients with COVID-19 and other pneumonias. The correlation analysis results showed that some textural features were positively correlated with WBC, and NE, and also negatively related to SPO2H and NE. Our results showed that radiomic features can classify COVID-19 patients and other pneumonias patients. The SVM model can achieve an excellent diagnosis of COVID-19.

## Introduction

The coronavirus disease 2019 (COVID-19)^1,2^ epidemic began in Wuhan, Hubei Province, China, in December 2019. Other cases in China and other countries soon followed. The main clinical symptoms are fever and fatigue. The respiratory symptoms are mainly dry cough and difficulty in breathing. In severe cases, acute respiratory distress syndrome, septic shock, and metabolic acidosis are difficult to correct and out of coagulopathy. Some patients have mild onset symptoms without fever. Most cases are mild to moderate with a good prognosis; a few patients are critically ill and die. The early manifestations of CT chest imaging^3–7^ include small-scale interstitial changes, which are evident in the lateral field of the lung. These further develop to multiple ground-glass opacity (GGO) and infiltration in the lung; lung parenchyma may be involved in severe cases.

Nucleic acid testing is the gold standard for the final diagnosis of COVID-2019 in non-invasive diagnosis. However, due to an inadequate supply of kits and complicated sampling methods, there are sure to be false negatives, which causes some patients to delay treatment and control measures. Besides, nucleic acid testing can only make a positive diagnosis. Recently, some researchers have proposed CT as a diagnosis standard for COVID-2019 to increase the detection rate. The advantage of CT is that it can make judgments quickly. However, CT also has its limitations: there is a certain degree of overlap with other lung infections, early infections may not have obvious lung imaging changes. Furthermore, considering biological safety issues and the risk of cross-infection, CT is still not the ultimate basis for diagnosis of COVID-19.

However, the diagnostic capability of artificial image reading is affected by factors such as seniority and experience; reading images with a large number is time-consuming. The existence of these factors inevitably has great subjectivity. In recent years, artificial intelligence (AI) has developed in many areas around the world. It is an effective tool for monitoring and responding to this global epidemic. The role of AI in combating COVID-19 is the earliest detection of potential infectious diseases. Many research teams have used deep learning to process reports from new cases, the Centers for Disease Control, and the World Health Organization, air routes, and other data.

Zhong Nan Shan’s team^8^ used an AI model [recurrent neural network (RNN)] to infer the epidemic trend of the epidemic in China. This effectively controlled the development of the epidemic. Recently, Zhongnan Hospital of Wuhan University worked with Shanghai United Imaging Intelligence Company to build and operate the United Imaging Cloud uAI platform. This uAI platform uses the VB-Net model^9,10^ to automatically segment and quantify the infected area in the chest CT scan and the entire lung. However, these AI technologies used deep learning technology, which required large data sets (tens of thousands of samples) for model training. Such large data sets are difficult to obtain and are expensive and time-consuming. In the early stage of the COVID-19 outbreak, the knowledge of this new disease was still very limited, and the data that could be collected was relatively small. Therefore, for relatively small data sets, traditional machine learning algorithms are usually better than deep learning methods. Therefore, this paper proposed a classification method based on the traditional machine learning method, i.e., a support vector machine, that used the radiomics features of CT chest images to identify and diagnose patients with COVID-19 and non-Corona Virus Disease 2019 pneumonias (other pneumonias).

## Material and methods

**Figure 1:**
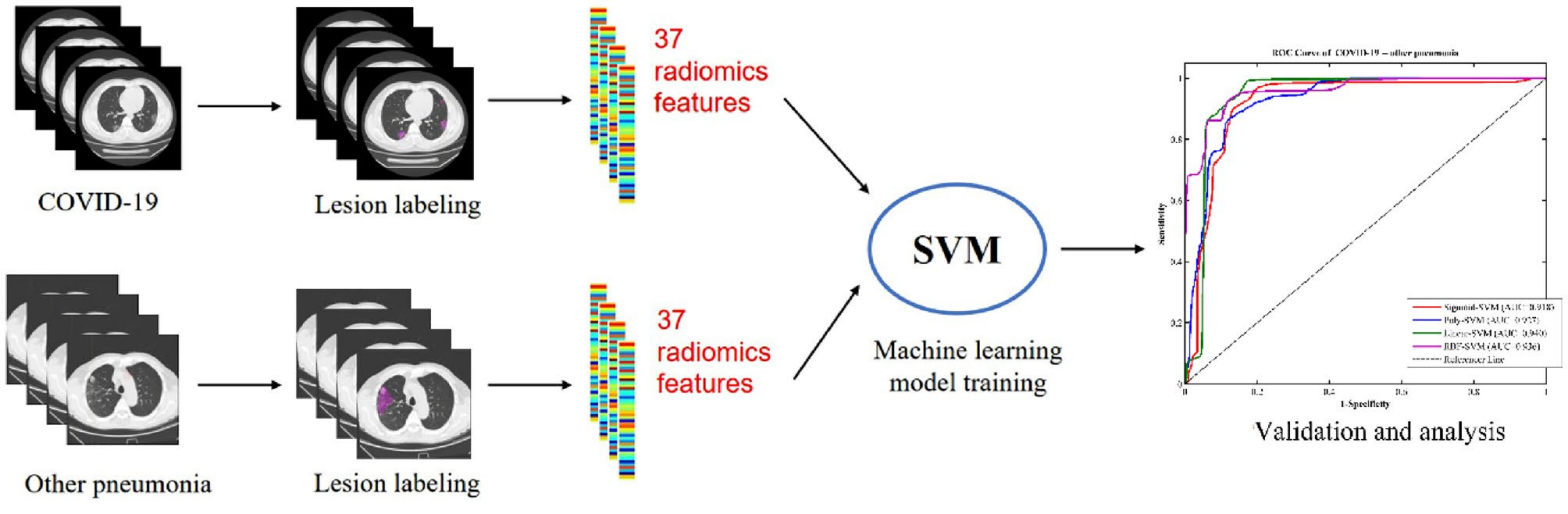
The flow of our proposed machine learning algorithm SVM with radiomics features for classification of COVID-19 and other pneumonias.

Here, we present a support vector machine (SVM) method for the classification of patients with COVID-19 and patients with other pneumonias via a radiomics framework. The workflow of our proposed method is shown in Fig. 1. First, lung infection areas (region of interest, ROI) in the CT images of each sample were artificially delineated. Second, thirty-two textural features and five histogram features were extracted from ROI data using a quantitative radiomics features model. Finally, a SVM classifier trained using such quantitative radiomics features from training data was used to distinguish COVID-19 patients and other pneumonias patients. All the methods involving human participants in this study were in accordance with the ethical standards of the institutional and/or national research committee and with the Declaration of Helsinki. The detailed methodology behind each step of the proposed method is described below.

### Participants and data acquisition

This retrospective study was approved and written informed consent was waived by the medical ethical committee of Guangdong Second Provincial General Hospital. Ninety patients who were confirmed by positive nucleic acid test of RT-PCR for COVID-19 (56 males, 34 females; mean ± standard deviation age, 45.36 ± 11.58 years) were recruited, and 90 patients with other pneumonias (Non-COVID-19 patients; 58 males, 32 females; mean ± standard deviation age, 46.54 ± 8.40 years) were recruited as a control group. All the other pneumonia cases were collected in 2017–2018.

Chest CT images of all participants were acquired using a 16-slice CT (Philips) from Guangdong Second Provincial General Hospital, Guangzhou, China. All chest CT images were acquired in about 2 min using a helical scan of the chest as follows: reconstruction slice thickness = 2 mm; reconstruction slice increment = 2 mm. The CT volume was composed of 98–165 slices with 512 × 512 pixels.

### Radiomics analysis

Radiomics textural analysis has been proposed since the early 1980s as a method for extracting relevant information representing tissue types from various medical images. Previous studies^11,12^ hypothesized that textural features can reflect heterogeneity within tumors, which is of great significance in cancer research. Textural analysis is a key component of radiology^13^.

A gray level co-occurrence matrix (GLCM)^14^ considers the arrangement of voxel pairs to calculate the textural index. GLCM is calculated from 13 different directions in 3D with a δ-voxel distance (‖$\overrightarrow{d}$‖) relationship between adjacent voxels. The index value is the average of the indexes in the 13 directions of the space (X, Y, Z). From this matrix, seven textural indices (homogeneity, energy, contrast, correlation, entropy_log10, entropy_log2, and dissimilarity) are computed.

The gray run length matrix (GLRLM)^15^ gives the size of the uniform run for each gray level. The matrix is calculated for 13 different directions in 3D (4 in 2D). Eleven textural indices are computed from this matrix: Short-Run Emphasis, Long-Run Emphasis, Low Gray-level Run Emphasis, High Gray-level Run Emphasis, Short-Run Low Gray-level Emphasis, Short-Run High Gray-level Emphasis, Long-Run Low Gray-level Emphasis, Long-Run High Gray-level Emphasis, Gray-Level Non-Uniformity for run, Run Length Non-Uniformity, and Run Percentage.

The neighborhood gray level difference matrix (NGLDM)^16^ corresponds to the gray level difference (8 in 2D) of a voxel and its 26 neighborhoods in three dimensions. Three textural indices (coarseness, contrast, and busyness) are computed from this matrix. The Gray Level Zone Length Matrix (GLZLM)^17^ provides information about the uniform zone size of each gray level in 3 dimensions (or 2D). Eleven textural indices are computed from this matrix: Short-Zone Emphasis, Long-Zone Emphasis, Low Gray-level Zone Emphasis, High Gray-level Zone Emphasis, Short-Zone Low Gray-level Emphasis, Short-Zone High Gray-level Emphasis, Long-Zone Low Gray-level Emphasis, Long-Zone High Gray-level Emphasis, Gray-Level Non-Uniformity for zone, Zone Length Non-Uniformity, and Zone Percentage.

**Figure 2:**
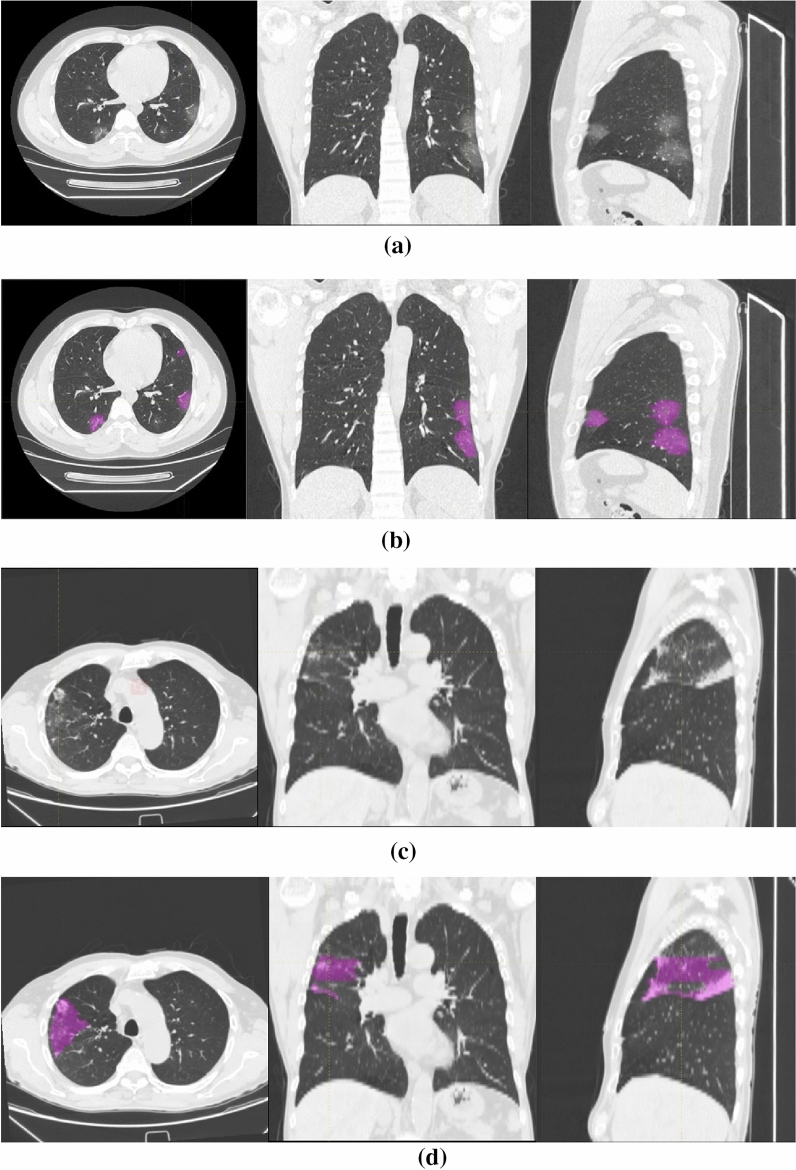
Image examples in different patient groups. From top to bottom: (**a**,**b**) a 35-year-old male with COVID-19 and COVID-19 lesion labeling (multiple ground-glass opacity, GGO); (**c**,**d**) a 77-year-old male with other pneumonia and other pneumonia lesion labeling (multiple patches and cloud floccules).

All textural analysis processes in this article were performed on the LIFEx (Local Image Features Extraction) platform^18^: three attending physicians with training in imaging delineated the lung infection area (region of interest, ROI) of each slice in the CT image of each sample. The senior physician was responsible for reviewing and modifying; finally, a three-dimensional ROI region was obtained in each CT image (Fig. 2).

The voxel size was then spatially resampled to 1 mm × 1 mm × 0.5 mm for a 3D ROI in each CT image of all participants. The initial voxel values were resampled into 256 grey levels and rescaled between mean-3 × Sd − mean + 3 × Sd of the ROI content, where mean and Sd are the mean and standard deviation of the voxels included in the ROI, respectively. Eventually, the 32 textural features described above were calculated from each ROI of the participants. We also built a histogram of each CT image and calculated five radiomics histogram features related to histogram skewness, kurtosis, and entropy.

### Diagnostic classification

This study used a machine learning method—support vector machine (SVM). The concept of SVM was first proposed by Vapnik and Cortes^19^ in 1995. It is based on the statistical VC dimension theory and the principle of structural risk minimization. It has many advantages in studies of small sample size with nonlinear and high-dimensional pattern recognition problems. The SVM finds a hyperplane that maximizes the distance between the two types of sample points closest to the hyperplane and the hyperplane.

After calculating textural and histogram features in each sample, we obtained a feature matrix (180 × 37) where 180 is the number of subjects (including 90 patients with COVID-19 and 90 patients with other pneumonias), and 37 is the number of extracted textural and histogram features. Using the feature matrix as input, SVM with different kernels (Linear, Radial Basis Function (RBF), Polynomial (Poly), and Sigmoid) was developed to train a machine learning model for classification in COVID-19 patients and other pneumonias patients. These classification models used 20 rounds of tenfold cross-validation method for training (70 COVID-19 patients and 70 other pneumonias patients). Single-shot testing results (accuracy, sensitivity, specificity, and area under ROC curve (AUC)) on an independent validation cohort (20 COVID-19 patients and 20 other pneumonias patients) were used as the final SVM classification performance. All machine learning processes for training and testing used PyCharm (http://www.jetbrains.com/pycharm/, JetBrains PyCharm Community Edition 2018.2.4 × 64) CT image analysis.

### Statistical analysis and correlation analysis

The demographic data for all participants were analyzed using SPSS 22. Differences in age between COVID-19 patients and other pneumonias patients were compared using the Wilcoxon rank-sum tests. Gender differences were assessed via chi-squared tests.

Nonparametric permutation tests estimated the statistical significance of average classification performance by determining whether the average classification performances exceeded the level of opportunity. The class labels of the training data were randomly ranked 1000 times before training, and the 20 rounds of tenfold CV procedure were repeated. The P value of the permutation test was defined as: P = (N_exceeds + 1)/(N_substitution + 1). Here, N_exceeds represents the number of times the permuted performance exceeded the one obtained for the true labels. The N_substitution represents the rounds of permutation.

In the COVID-19 patients, correlation analysis was also conducted to determine whether the textural and histogram features correlated with the laboratory test index of blood, i.e., blood oxygen (SPO2H), white blood cell count (WBC), lymphocytes (LYM), neutrophils (NE), C-reactive protein (CRP), hypersensitive C-reactive protein (hs-CRP), and erythrocyte sedimentation rate (ESR).

## Results

### Demographic data results

Demographic data results shows that there were no significant differences between the COVID-19 patients and the control group in terms of age (P = 0.61) and gender (P = 0.83).

### Diagnostic classification results

**Figure 3:**
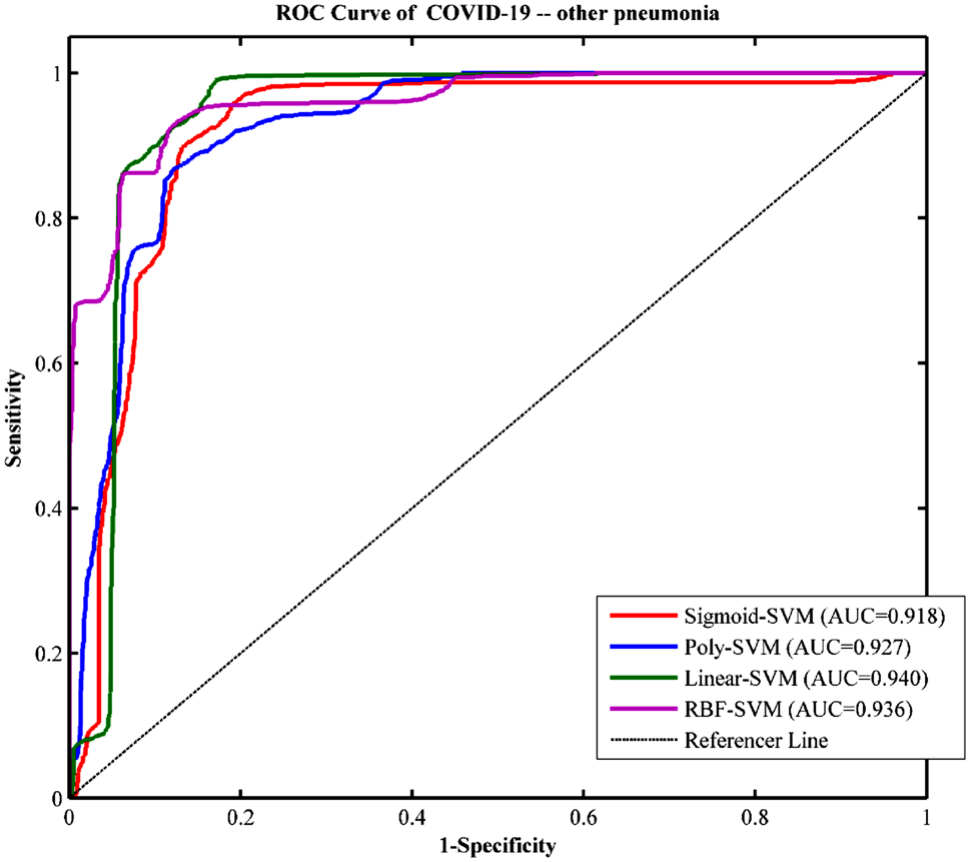
ROC curve of a SVM model based on different kernels for classification of COVID-19 and other pneumonias.

The textural and histogram features led to a feature matrix of (180 × 37), where 180 is the number of subjects (90 COVID-19 patients and 90 other pneumonias patients), and 37 is the number of extracted textural and histogram features. Using the feature matrix as the input, SVM with different kernels (Linear, RBF, Poly, and Sigmoid) was developed to train machine learning models for classification in COVID-19 patients and other pneumonias patients. There were 20 rounds of tenfold CV procedures for training (Fig. 3). With single-shot testing on an independent validation cohort, the ROC (receiver operating characteristic) curve of all SVM models prepared based on these different kernels had good performance. The Linear Kernel-SVM model showed the best discrimination with an accuracy of 89.83%, sensitivity of 94.22%, specificity of 85.44%, and AUC of 0.940 (all P < 0.001). RBF Kernel-SVM model also showed good discrimination with an accuracy of 88.53%, sensitivity of 87.50%, specificity of 89.56%, and AUC of 0.936 (all P < 0.001). The results suggested that the textural and histogram features between COVID-19 patients and other pneumonias patients were highly distinguishable, and the machine learning method achieved excellent classification effects. This contributed to the diagnosis of COVID-19 and other pneumonias. In addition, the kappa scores of inter-observer agreement were calculated and the lowest kappa score between two observers was 0.816, which showed good reproducibility for model performance on ROIs drawn by multiple observers.

We also performed our proposed method using GLCM, GLRLM, NGLDM GLZLM and Histogram features as model inputs respectively. As seen in Table 1, with different features, all the SVM models reached accuracy higher than 75%; the classification effect with GLCM or GLRLM as model input is better (GLCM is best with accuracy as high as 86.67%, sensitivity of 82.22%, specificity of 91.11%, and an area under the curve of 0.913), while GLZLM is the worst. The results suggested that GLCM features between COVID-19 patients and other pneumonias patients were the most distinguishable.

**Table 1:** The classification results of COVID-19 and other pneumonias with different features (GLCM, GLRLM, NGLDM GLZLM and histogram features) as model inputs, respectively.

Feature	Accuracy (%)	AUC	Sensitivity (%)	Specificity (%)
GLCM	85.95	0.915	88.90	83.00
GLRLM	83.80	0.900	92.10	75.50
NGLDM	80.65	0.890	86.60	74.70
GLZLM	79.25	0.876	86.10	72.40
Histogram	81.65	0.895	91.20	72.10

### Correlation analysis results

In the COVID-19 group, correlation analysis (multiple comparison correction—Bonferroni correction, P < 0.05/7) was also conducted to determine whether the textural and histogram features were correlated with laboratory assays. As seen in Table 2, the correlation analysis results showed that some textural features were positively correlated with WBC, NE, and also negatively related to SPO2H and NE. However, it should be noted that all significant correlation results are low correlation.

**Table 2:** Correlation analysis results between the textural features and the laboratory test index of blood.

Textural feature	SPO2H		WBC		NE		CRP	
	r	P value	r	P value	r	P value	r	P value
GLRLM_LGRE					0.310	0.003	0.286	0.007
GLRLM_LRLGE					− 0.316	0.003		
GLZLM_SZE	− 0.344	0.001	0.307	0.003	0.337	0.001		
GLZLM_LZE					0.315	0.003		
GLZLM_LGZE					− 0.310	0.003		
GLZLM_SZHGE	− 0.350	0.001	0.310	0.003	0.314	0.003		

## Discussion

In this study, we built an effective COVID-19 diagnosis system based on a traditional machine learning method. Our purpose was to examine whether traditional machine learning could be useful in the diagnosis of patients with COVID-19 and non-COVID-19 pneumonias (other pneumonias). We chose the SVM approach over deep learning because deep learning often requires relatively large training samples to avoid over training and because of a small sample size in this study—traditional machine learning methods can sometimes achieve better performance.

One of the challenges in COVID-19 diagnosis is its different diagnosis with other pneumonias. We collected 90 samples with COVID-19 and 90 samples with other pneumonias as a control group. GLCM, GLRLM, NGLDM GLZLM and Histogram features were calculated from the pneumonia lesions in the CT images of each sample. Respectively, using these five categories of features as inputs, our proposed method also achieved good classification effects. As seen in Table 1, the SVM model with GLCM features achieved best classification effect: accuracy of 85.95%, sensitivity of 88.90%, specificity of 83.00%, and AUC of 0.915. It suggested that GLCM were the most distinguishable features between COVID-19 patients and other pneumonias patients. In addition, NGLDM, GLZLM and Histogram had high sensitivity and relatively low specificity. Combined radiomics features (32 textural features and 5 histogram features) for SVM model training could distinguish COVID-19 from other pneumonias with accuracy of 89.83%, sensitivity of 94.22%, specificity of 85.44%, and AUC of 0.940. It was noted that combining different features helped to improve the classification effect, which proved that these features provide different information and can be useful for the diagnosis of COVID-19. The results showed the potential of our proposed method in clinical practice by aiding the radiologist in proper diagnosis.

According to “COVID-19 Pneumonia Diagnosis and Treatment Plan (Trial Version 8),” the total number of WBC in the early stage of COVID-19 is normal or decreased. The early warning indicator for medium and critical adult patients with COVID-19 is a progressive increase in CRP; in addition, symptoms of poor breathing and dyspnea will occur due to the virus invading the lungs, and hypoxia will result in decreased SPO2H. As seen in Table 2, we found that some textural features were positively correlated with WBC, and also negatively correlated with SPO2H. This explains why textural features are discriminative in the two groups of COVID-19 patients and other pneumonias patients. Although the correlation result between CRP and GLRLM_LGRE was significant, the correlation coefficient was less than 0.3, and the degree of correlation was extremely weak, which could be regarded as irrelevant. We also showed how some textural features were correlated with NE; however, the clinical manifestation of NE in COVID-19 patients remains unclear since this disease is new. Knowledge about its appearance is continually updated. Future research will validate this result.

Compared to other radiomics-related literature on AI-assisted diagnosis and classification of COVID-19 published in recent months, there are several limitations to our study. First, the data set and the radiomics features in our study is limited. Second, the outlines of lesions (ROI) in CT images were manually labeled layer-by-layer, which was time consuming (approximately 15–25 min for each sample). Recently, many related studies^20–22^ adopted deep learning methods, such as CNN, to automatically segment the regions of interest in the lung, which could avoid the problem of manual outline. However, deep learning method requires a huge data set as inputs, which also requires us to collect more cases in the future. Third, our study was lack of feature engineering. Liu et al.^23^ and Qiu et al.^24^ used the least absolute shrinkage and selection operator (LASSO) algorithm to select the optimized features. This can improve the performance of model and reduces the complexity/training time of model. Forth, this single country study might have racial bias. Fifth, we also assessed whether COVID-19 pulmonary lesions were mild or severe^23,24^ using our proposed method. In the classification of mild cases and severe cases, our proposed method achieved a classification accuracy of 71.67%, sensitivity of 83.57%, specificity of 48.33%, and an area under the curve of 0.827. This result was not ideal because of the poor specificity. In future experiments, we will further study the severity of COVID-19 pneumonia by using other AI and radiomics methods to improve the effect.

## Conclusion

Future work will solve the limitations above by collecting more cases, using deep learning to achieve an automatic outline of pneumonia lesions, performing feature extraction and feature selection and adopt other AI methods. In summary, despite these limitations, our results showed that radiomics features can classify COVID-19 patients and other pneumonias patients. The SVM model can achieve an excellent diagnosis of COVID-19.
